# An Essay upon the Medical Properties of Plants Compared with Their Exterior Forms and Their Natural Classification

**Published:** 1828-07

**Authors:** 


### REVIEWS AND BIBLIOGRAPHICAL NOTICES.

—

Art. IV.—*Essai sur les Proprietes Medicales des Plantes, comparees avec leurs formes exterieures et leur classification naturelle: par* M. Aug. Pyr. De Candolle, *Professeur de Botanique aux Facultes de Medicine et des Sciences de l'Academie de Montpellier, Professeur-honoraire a l'Academie de Geneve, Correspondant de l'Institut, des Academies Royales de Sciences de Munich, Turin, etc. Seconde edition, revue et augmentee. A Paris,* 1816, *pp.* 397, 8*vo.*

*An Essay upon the Medical properties of Plants, compared with their exterior forms and their natural classification. By* A. P. De Candolle, &c.

About half a century ago, when the "American Philosophical Society, held at Philadelphia," was almost the only association of philosophers in the new world, its leading members were greatly devoted to practical Astronomy. The objects on which they occupied themselves, were not less within the observation of the astronomers of Europe than of the U. States, while the former were far better supplied with instruments than the latter; and, consequently, were able to make exacter contributions to science. At the same time America was undescribed—her natural history unwritten. Her geological structure had not been exposed to view; her mineral treasures, like the precious metals of the miser, lay buried in the earth, and the countless variety of plants which shot from its surface, in unrivalled beauty and luxuriance, were by them left to bloom unheeded in the wilderness. Such was the misapplication of American intellect. Better conceptions at length arose; and it was seen, that while the Heavens could present nothing new to *our* philosophers, the Earth where they lived was prolific of novelties, and might be made to yield an opulent harvest. *It*, then, became an object of study; and the very city which was the cradle of American

Astronomy, has since become still more distinguished for its successful cultivation of Natural History and Medicine.

Since the era of which we have spoken, the West has been explored and settled. Men have encamped beneath its overshadowing forests, which have been thinned out by the axe of industry; its beautiful shrubs have been eradicated; its tribes of flowering and medicinal herbs trodden under foot, and in part made to disappear, like the wild animals which fed upon them. Population has thickened—organized society has begun to appear—the germs of useful knowledge have been planted. Medicine, the inseparable attendant on civilization, has been introduced, and its cultivators are to be found in every inhabited spot. They are surrounded by the peculiar plants which flourish in the bosom of a newly discovered continent; and, still, most of them are as inattentive to the science of Botany, as the earlier philosophers of America were to its natural history at large. Our present object is to point out this omission, and to recommend the cultivation of Botany, to our medical friends of the West.

Physicians ought to recollect (and they might be permitted to recollect it with pride) that the very name of their profession, is almost a synonym for nature; and that this title was appropriated to them, under a clear perception of the objects which should engage their attention. They are not called upon, however, to study all nature; this would be a fatal diffuseness. They must concentrate themselves upon a spot, but this, on the other hand, should not be too small; for the examination of a part can never make us acquainted with the whole. The proper study of the profession is organized nature, and this comprehends the vegetable as well as the animal kingdom. Between these two departments there is more intrinsic similarity than many persons suppose. The former, it is true, is far more complicated than the latter; but still the differences between them are to be found in their organs, rather than their constitutions. They are both highly vascular, and circulate fluids which undergo a great variety of transformations, affording many results which are almost identical in the two classes. Their growth is apparently on the same principle; and they, alike, become old and perish. Finally, plants have their diseases, which greatly resemble those, which, in animals, we denominate organic. Thus the principle of physical animation and that of vegetation, seem to be mere varieties of the same power; and he who would comprehend its nature, should trace it throughout all its operations.

We might rest the question upon this reference to general physiology; but an equally conclusive argument in favour of Botany, may be raised by referring to therapeutick medicine. A large proportion of our most important medicines, belong to the vegetable kingdom; and although a physician may prescribe them, successfully, without an acquaintance with Botany, it is undeniable, that had not the vegetable kingdom been studied, scientifically, our Materia Medica would at this time, be far from that perfection which it boasts. Many of the greatest botanists have been physicians—of which we may cite Linnæus, Boerhaave, Withering, Darwin, and Barton. If the study of Botany has been necessary to the advancement of practical medicine in past times, it will be equally so in future; and, if it recommend itself to the profession generally, it still more forcibly recommends itself to the profession in the United States; and, especially, in the West and South. It is lamentable to observe the mistakes which have found their way into certain fashionable works on the Materia Medica; compiled or reprinted in this country, by gentlemen learned in every science, but that on which they undertook to write. In these reprints and compilations, it is perfectly easy, to place the finger of criticism on every botanical interpolation; for it bears the stamp of error or imperfection. Such productions add but little to the stock of professional knowledge; and still less to our national character. We have heard it said, however, that physicians have not time for the cultivation of any branch of natural history. This is not the fact. They fail, not from want of time, but from want of application; and this brings us to say, that the physicians of this country, both before and after graduation, are in general deficient in application, to every thing, but the practical duties of the profession. It is undeniable that the majority of them enter upon those duties with a *minimum* of elementary knowledge, (excluding the rudiments of natural philosophy, mineralogy, zoology, botany, and, even, meteorology) and too often read little else, than the public journals, and an occasional compilation on practical medicine, ever afterwards. This is the great American failing. To this idleness (seeing that in books of science things should be called by their right names) we may fairly ascribe the imperfection of most of our contributions to medical science, when compared with those of our European brethren, whom experience has taught to rely, not on the pretended inspiration of genius, nor even the multiplicity of opportunities, so much as unabated diligence. This in fact is the *sine qua non* to distinction, in the medical profession; and, indeed, in every other. The proper time for a physician to cultivate Botany, and other branches of natural science, auxiliary to medicine, is the first few years after his graduation. He ought, indeed, to acquire the first idea of those sciences during his novitiate. Our colleges, however, in their wisdom have seen fit to dispense with all such attainments, with the exception of Chemistry (which is often studied most superficially) and few students are disposed to acquire more than the laws of the college prescribe, as indispensable. No young physician has, nor ought to have, much professional occupation. Every case should be a study for him; and he cannot, therefore, attend to as many in the same time, as the man of age and experience. Hence the correctness, on the part of society, of a slow extension of its confidence and patronage. During this period of probation, he has ample time for the cultivation of Botany, and indeed of the other sciences which lend a helping hand to Medicine; and to it we would earnestly direct the attention of the younger members of the profession in these western states. The fixed habits of our elder brethren cannot of course be altered.

The advantages which result to the individual from the study of Botany, are not limited to its connexion with medica science. Few studies contribute so much to strengthen the faculties of observation and comparison, as that of Botany. It is a science of visible relations; and every practical botanist, must of necessity, become a good observer. The habit of nice discrimination thus acquired, is to a physician invaluable. Without it, the most comprehensive views in the philosophy of medicine, and the greatest erudition, will not preserve him from perpetual mistakes at the bed side of the sick; where, beyond every other situation, the consequences of error are most fatal.

But we must come to the work before us. Its object is clearly set forth in the title page; and will be, generally, acknowledged to be of substantial interest to the profession. We know of no book in our language of the same kind; and have, therefore, although it is not a very recent production, thought it worthy of being introduced to the notice of our readers.

The fundamental propositions of M. De Candolle's Essay are not new, but he has the merit of having illustrated them by a greater number of facts than any other person; and for this task, his manner of regarding and prosecuting the science of Botany had peculiarly fitted him.

In the Linnæan system, plants are distributed into classes, orders, and genera, by a reference to their fructification only; but it is well known, that many of the classes and orders of this method are artificial. A natural classification brings together such plants as resemble each other in all their parts. Such a method was long since attempted; and although attended with great difficulties, has been brought to higher perfection, than could, at first, have been anticipated. Among the distinguished labourers in this department, is M. De Candolle. He is, indeed, the author of one of the latest systems of natural classification, which is that adopted in the Essay now under review. By this system nearly all known plants are divided into 150 groups, or orders; each of which is subdivisible into genera or families. Now, the essential proposition of the work which we are about to present to the notice of our readers, is, that plants which resemble each other in their external or botanical characters, also, resemble each other in their chemical composition and in their action upon the living body. From whence we derive the corollary, that the medical properties of one species of a natural group being given, the qualities of all the rest may be inferred, at least with considerable probability.

In the first part of his Essay, M. De Candolle undertakes to develope the general proofs of the analogy which exists between the forms and the properties of plants, and the rules by which the comparison should be conducted. The first chapter is divided into several sections; the first of which embraces the proofs deduced from theory; in which our author insists, that from principle we should expect to find the virtues of plants analogous to each other, when their botanical characters are analogous. The second section sets forth some of the proofs which are furnished by observation. If the different species of a natural family of plants possess a similar composition, and have similar properties, we might expect brute animals and insects, which have an instinctive fondness for a particular species, to extend their depredations to all others possessing analagous characters, provided they possess analogous constitutions, but not otherwise. Now observation informs us, that, in general, an animal which is prone to subsist on any particular species, will in the absence of that species, select others which are related to it in botanical characters; a fact which conclusively indicates a similarity in composition and qualities. Thus,

"The ox leaves untouched the whole of the labiated plants, and all the veronicas; and the horse refuses nearly all the cruciferous vegetables. Both these animals, the sheep, the hog, and the goat, scarcely eat any of the solanaceous plants, while they devour, with avidity, the grasses, the leguminous vegetables, and those with compound flowers. Animals naturally limited to a single kind of nourishment, sometimes extend their ravages to all the species of the same family. The insects might furnish a thousand examples of this kind. Thus the *Curculio Scrophularia* of Linnæus, the *Cynips Rosæ*, the *Psylla Juncorum*, the *Curculio Rumicis*, the *Cynips Salicis*, &c. attack many species of the genera, which bear these names, and some of them subsist indifferently upon all. For example, the silk worm is nourished, in different countries, with the leaves of the white, black, red, Indian, and Tartarian mulberry." pp. 21, 22.

In the third section we have a brief statement of the proofs drawn from experiment; which the author justly regards as more conclusive, than those furnished either by reasoning or observation. The following paragraph will set forth his views on this subject.

"If," says he, "we inquire into the history of the Materia Medica, we find that a great number of medicines, even the most active, which in the infancy of the science, were respectively regarded as the product of a single plant only, have been since found in many kindred species. Thus the cinchona bark is derived from all the true species of that genus; rhubarb from nearly every species of *rheum*, opium from many different kinds of poppy, semen—contra (a worm powder) from a number of species of artemisia, and turpentine from the greater number of pines. Further, the best accounts inform us, that tragacanth is afforded by many spinous *astragali*, as gum arabic flows from many acacias; the roots of a kind of violet, growing in different places, are found to be emetic, and, I think I have rendered it probable, in a Memoir, that the anthelmintic property of the Fucus Helminticorton is common to a number of the Ceramiums. Many species of the same genus, afford medicines so similar, that before knowing their history we had united them under one name.

"There are others which, being better known to us because they are indigenous to France, have always been regarded as endowed with the same virtues; thus all the mallows are emollient, the scurvy grasses are antiscorbutic, the gentians febrifuge, the monkhoods and the hellebores, caustic and dangerous, the euphorbiæ acrid and purgative," &c.—p.28.

We might extend our quotations; but, the knowledge of every reader, will supply him with similar facts. It will, also, present him with many apparent exceptions to the rule; exceptions which our author recognizes, and proceeds to consider and explain away.

To accomplish this, he investigates, and attempts to settle the rules of comparison, between the properties and the external forms of plants; and this makes the subject of the second chapter. The first section treats of classification. Many naturalists speak of organized beings as constituting a chain. Our author objects to this manner of considering them. In the first place, "Nature,' says he, 'does not conform to our books. Each being is placed among a certain number of other beings, with which it has more or less affinity; and the best means we possess of communicating an idea of this relation, is to represent all natural objects, not in a series, but as placed on a geographical chart. The species correspond to the towns, the genera to provinces, the orders to empires, the classes to continents, and insulated species to islands. If such a chart were completely executed, and laid before us, the first thing that would strike us, as in a true geographical map, would be, that in certain empires or certain provinces, the towns would lie very near each other, while in others they would be widely separated. This separation would indicate one of two things; either that there were intermediate species yet unknown, or that nature, in the order of being, had really left vacant spaces, here and there, as, upon the surface of the earth, she has created unihabitable morasses and deserts. Here we have then a first cause of uncertainty— in the unequal distances from each other, of the species in the different genera or the different natural families. We ought, therefore, no more to be astonished, that the grasses, the mallows, and the labiate and cruciform plants, very closely resemble each other in properties, while the *caprifolaceæ*, the *rutaceæ*, the *urticæ*, and some other families, present anomalies, than we should be surprized, in the social order, to see a very populous and highly civilized country, exhibit uniformity of manners, while regions almost uninhabited, or divided by rivers or mountains, offer striking differenees in the character of their people.

"Secondly. As in political arrangements we sometimes unite a detached city or a small island with the nearest province; so in the order of nature to avoid a multiplicity of divisions, the botanists attach to a genus or an order, a species which differs from the rest in its organization. In these cases if the properties differ, it is because the organization is different, and the exception confirms the rule.

"Thirdly. It frequently happens, that some plant which varies widely in its properties from the family or the order in which it has been placed in our systems is, subsequently, found, in reality, to appertain to some other."—pp. 33—36.

Passing by the examples with which our author illustrates this proposition, we come in the second section, to a comparison with the organs of plants. The section opens with a distinction between the general and the particular properties of plants. The former result from a mixture of the whole of the latter, which only, deserve consideration, in this inquiry.

"It is evident,' says he, 'that we ought to compare each organ of a plant with the corresponding organs in other plants, and, in this comparison, the more we descend into detail, the nearer we approach to exactness. Thus how many plants do we not see, which possess very different properties in their different parts! M. Jussieu has offered us a fine example of the utility of this exactness, in showing us, that in the seeds of the Euphorphiæ the integuments are sweet and healthy, while the inner parts are acrid and strongly purgative.—Guided by this principle, we shall not make the mistake of comparing the potatoe with the berries of the other species of Solanum, nor the roots of the carrot with the leaves of the hemlock. Thus we have already weakened one of the strongest objections against the analogy for which we have insisted; and it will be still further reduced if we follow out the consequences of this comparison of the organs.

"First. It is evident that if some plants of a family enjoy a particular organ, which is either wanting, or very little developed, in the other plants of the order to which it belongs, we ought not to be astonished if the properties of this organ are not found in those plants. Thus if the pulp of the Vanilla enjoys aromatic properties which are not found in the family of the Orchideæ generally, do we not perceive the cause, by observing, that the pulp which envelopes the seeds, is entirely wanting in the other genera of the order? Is not the same pulp, soft and laxative in the the cassia and tamarind, wanting in the greater part of the leguminous plants? Examining this subject more closely, we find certain organs, which, if we may be allowed the expression, are accidental; and which have the same properties, whenever or in whatever family, they may be developed. Thus the tubers which are attached to certain fibrous roots, and which should be carefully distinguished from a simple swelling of the body of the root, contain a mild and nourishing fecula, as we see in the potatoe, Jerusalem artichoke, sweet potatoe, drop wort, &c. &c.

"Secondly. If, on the contrary, the useful properties appertain to some organ, essential to the family, we find that these properties offer but little variation. Thus the farinaceous perisperme of the grasses, is highly nutritive and of an agreeable taste. The seeds of the umbelliferous plants, all of which have small vessels filled with essential oil, are, the whole of them, stimulating and aromatic &c.

"Thirdly. If the same properties appear to be found in similar plants, but in different organs, we may, it seems, to me, find the cause of this anomaly, in studying with greater care the relations of these organs. This subject, which belongs to vegetable anatomy, requires developments of considerable length, and a degree of knowledge which perhaps the botanists have not yet acquired; I will, therefore, content myself with citing such examples as will enable us to perceive its utility. When we examine the series of monocotyledonous plants, we are surprized to see the bulbs of the liliaceæ furnish fecula, almost like the stems of the palms; while other bulbous roots, have a cathartic property, analogous to that afforded by the juice of the stem and leaves of the aloe. This resemblance between the stems and bulbs, which may appear an exception to the rule which I have attempted to establish, is, on the contrary, in my opinion, a confirmation; for vegetable anatomy proves, it appears to me, that the bulb ought not to be assimilated to the roots but to the stems: I will explain myself."—pp. 37—40,

Our author here proceeds to give his reasons for this opinion, which are ingenious if not conclusive: but as the subject is purely botanical, we shall pass over, and proceed to the next section, in which he enquires into the influence which soil, climate, and other causes, exert, in modifying the properties of the different species of a family. The following extract will show his manner of considering this subject:

"M. Theodore de Saussure has shown us, that this influence (that of the soil) extends much further than we had thought, by having remarked, that the same plants growing in granite or in calcareous soils, offer remarkable differences in their chemical composition, and in their nutritive properties. The attention of physiologists has been excited upon this subject too short a time, to enable us to draw from it many direct consequences; but we know more of the influence of the soil in other respects: thus in certain families we see the properties of the same plant vary a great deal, according as it has grown in a dry or a humid situation; the *Heracleum Sphondylium*, a common plant in our meadows, and which different domestic animals eat without inconvenience, becomes sometimes poisonous, when it grows in a very wet place, or when the year is too abundant in rain. We see in the same manner, that the celery which is collected in marshes, where it grows naturally, is acrid, nauseous, and poisonous, while it becomes sweet and proper for nourishment when it is cultivated in a dry soil. If the same species of umbelliferous plants offer such anomalies, ought we to be surprized at seeing the other plants of that family acquire, in general, a property more or less poisonous, when they grow in wet places, as we see in the *Phellandrium Aquaticum, Cicuta Virosa, Œthusa Cynapium, Œnaothe Crocata*, &c., while, on the contrary, those which grow in dry places, exposed to the sun, are more or less mild, aromatic, and stimulating: such are—*Angelica Archangelica, Coriandrum Sativum, Anethum Fœniculum*, &c. According to the same observation, ought we to be astonished, that all the poisonous umbelliferæ are natives of cold or temperate countries, while those which grow in warm countries are aromatic and usefully employed as stimulants."—pp. 43, 44.

In like manner our author states the influence of elevation, of light, of the season of the year, and of the age of plants, upon their properties; the whole of which, we should be pleased to transcribe, but our limits do not permit.

The fourth section is chemical. In the first place, the diversities, in quality, which similar plants present, from a mere variation in proportion, of their ingredients, may be very great. Thus, for example, analogous plants may abound in the gum-resins, but the relative qualities of these principles may be constantly varying; and in the roots, which contain fecula mixed with an acrid juice, such as those of the genus Arum, the latter may exist, sometimes, but in very minute quantities, or even be entirely wanting. In the second place, among the immediate materials of vegetables, there are some which exist successively, in several different states. Thus mucus changes to sugar, and this again seems to furnish the elements of fecula, the fixed oils are transformed into wax, and the volatile, into resin, &c. So that if the two species were compared in different stages of this transformation, they might present great apparent differences, when in reality they possessed great similarity. In the third place, when we see very different plants apparently affording the same principles, which is contrary to the theory, we shall often find, upon closer inquiry, that the resemblance, in properties, is not, in reality, so great as we at first supposed.

"Thus, although the bitter taste denotes, in general, analogous qualities, in the plants which are endowed with it, still this is not rigorously true, except when it relates to vegetables of the same family. The bitter and deleterious principle of the Strichneæ; the bitter and poisonous principle of the Menisperma; the bitter and salubrious principle of the Gentianæ; the bitter and animalized principle of the Armoises; the bitter and resinous principle of the Magnoliaceæ; the bitter principle, so eminently soluble in water, of the Simaroubeæ; the bitter principle of the Angusturæ, which gives with iron a yellow colour; that of the Chamadrys, which precipitates iron of a green colour; finally, that of the Quinquina, which precipitates the nut gall, and dissolves equally, in water and alcohol;—all these different bitters, I say, are different substances, and cannot be, rigorously, compared with each other. We arrive at the same result, if we compare, in the same manner, the astringents, owing either to the gallic acid, found in the Ratanhia, the tannin existing in the Acacias, or the union of these two ingredients, as they exist in different plants, &c.

"These important considerations furnish us with an answer to one of the strongest objections, that made by M. Halle, to the analogy between forms and properties, viz. that the same properties are found in very different families. This assertion may be strictly true, when made of determined properties, and relative to chemical materials, perfectly pure and identical in all vegetables; thus the slight modifications which we observe in the feculas, fixed oils, gums, ligneous matters, &c., are of little importance; but when the assertion is made of principles less uniform in their composition, and not so generally spread in the vegetable kingdom, the distinction of families will be found to display all its importance; thus we know, that the bitters and astringents, in the same family, are *only* similar to each other; all physicians know very well, that the action of narcotics, obtained from different families, is far from being the same; thus we find a narcotic principle in the *Papaveraceæ*, the *Chickoraceæ*, the *Borragineæ*, the *Drupaceæ*, *the Solanaceæ*, the *Nymphæaceæ*, &c.; but they are different from each other, and are no more to be confounded in the practice of medicine, than in the classification of the botanists; the application of the theory of natural relations to the Materia Medica, may even be useful, in many cases, as a means of classing with greater exactness, the varieties which present themselves in the same series of medicines: thus, not to depart from the example already cited, it seems to me, that a classification of narcotics, founded upon the natural families of plants, would be at least as exact, as those which are found in most treatises on the Materia Medica."—pp. 49—51.

The fifth section treats of the influence of pharmaceutic manipulations, in modifying the active properties of the ingredients of plants; and points out the necessity of making those processes exactly uniform, when we propose to use their results in estimating the relation, between natural affinities and medicinal qualities.

The sixth, discusses the modifying agency of the mechanical and some accidental properties of plants, on our estimate of their essential medical virtues. Of this section we shall translate a single paragraph, which contains a suggestion that will perhaps be new to most of our readers, and may serve as a specimen of the author's views.

"Thus when the dog, urged on by instinct, eats the dog's grass (Chiendent) to cause vomiting, we ought not to count that herb among the emetics, since to all appearance, it only produces this effect upon the dog, because that animal, furnished but with cutting teeth, cannot grind it; and in swallowing it half masticated, experiences an irritation in the œsophagus, which excites vomiting, as it is occasioned in man, by tickling the fauces."—p. 54, 55.

In the last section, our author speaks of the errors which the profession have committed, in their therapeutick observations; and attempts to show that great inaccuracies may creep into our remarks on the modus operandi of medicines; in consequence of which we may be led to disbelieve in fixed relations, between botanical affinities and medical properties, when they really do exist. The great causes of error are two:

"1. We often designate under two different names, and range in different classes, medicines, the action of which upon the animal fibre, is really similar; and this error depends on two causes; first, applying two different medicines to different organs, and then comparing their effects, instead of applying them to the same organ; secondly, comparing their effects when administered in very different portions—one being given in a large and the other in a small quantity—variations in the dose which must afford striking differences of effect.

"2. Having considered how, either by diversity in diseases, or in the doses of medicine, the effects of those which are really analogous in virtues, may be very different, it remains to examine the last source of error which I shall notice. It is this, that substances really different, sometimes produce similar effects. There is scarcely any class of medicines, in which we cannot find examples of this phenomenon. Thus although the increased flow of urine from diuretics, seems to be a very simple effect, it is, nevertheless, produced in three different modes;—by the mere augmentation of the mass of liquids in the body; by a special stimulation of the kidneys; or by increased excitement of the whole system. The succulent fruits, the digitalis and the squill, appear to me to offer examples of these three classes of diuretics."—p. 61, 62.

This example, although in some respects exceptionable, may serve to explain our author's meaning; and, desiring to close our account of this part of his book, we shall conclude with the following extract:

"Here terminates the first part of this work. We have traced out the principal rules which the present state of Botany, Chemistry, and Medicine can present, to govern us in comparing the properties of plants with their natural classification; and each of them, instead of augmenting the number of exceptions, has furnished, on the contrary, a solution of many anomalies; a circumstance which, if I do not deceive myself, goes far towards establishing the truth of the theory."—p. 63.

Having delivered what may be called the theory of his subject, our author comes, in the second part, to the practice. This is by far the largest portion of his book. He regards all plants as divisible into a number of natural groups or orders, which, in another work, he has arranged in a series of his own. Each of these groups he now takes up, separately; and examines, whether its properties are equally uniform, with its botanical characters. This is obviously an undertaking of great magnitude; and requires, for its perfect execution, a far more intimate knowledge of the botanical relations, chemical composition, and therapeutic qualities of plants, than we have yet attained. Hence, in travelling through the series, we perpetually meet with natural families, the virtues of which are so imperfectly known, that they can only be indicated, as proper objects of future inquiry. In others, the properties of the species correspond beautifully with the natural characters, and hold out to the cultivators of this interesting branch of science, a prospect of ultimate success, that should animate them to continued exertion.

Of this part of our author's work, we shall translate and present to our readers, an account of as many natural orders as our limits will permit, selecting those which will be intrinsically interesting to the profession, rather than such as are best calculated to demonstrate the reality of the connexion between botanical affinities and medical properties.

"3.—MAGNOLIACEÆ.

"Although the family of *Magnoliaceæ* is composed of a small number of species, it merits the attention of physicians, by the intensity of their sensible qualities.

"The bark of all the species, possesses a bitter taste, without astringency, combined with a pungent and aromatic quality. The last property is, especially, remarkable, in the different species of the genus Drymis, which indeed, suggested the name;* such as the *Drymis Winteri*, celebrated under the name of *Winter's Bark*, but which must not be confounded with the *Winterana Canella*, which belongs to the family of Meliæ of Jussieu; the *D. Granatensis* of Santa Fe, the *D. magnoliæfolia*, named *Canelo* in Chili, and two other species, imperfectly known, indigenous to Mexico and Brazil: The barks of all these species are slightly bitter, very sharp, pungent, and aromatic; and are employed with success as stimulating tonics, and stomachics, in dyspepsia and in certain fevers. The same physical qualities and medicinal properties are found in the bark of the *Melambo*, lately introduced into European practice, and which appears to me, evidently to come from a Drymis or some kindred genus. This bark contains neither tannin nor gallic acid, but a volatile, aromatic principle, and a great quantity (nearly one fourth of its weight) of a bitter and resinous matter. The aroma of the bark of the Drymis is found, although less intense, in the bark of the Illicium, in that of the *Magnolia glauca*, *grandiflora*, and *auriculata*, finally, though feebler still, in that of the *Liriodendron tulipifera*. In the two latter genera, the bitter principle predominates; and they are generally employed in America as tonics and febrifuges. Several of them, as the *M. glauca*, and, especially, the *L. Tulipifera*, (yellow poplar) are used in the United States of America, as substitutes for the Peruvian bark.

"The analysis of the bark of the yellow poplar, published by M. Tromsdorf, demonstrates, as in the case of the Melambo, a total absence of tannin and gallic acid, with the presence of a considerable quantity (one eighth of its weight) of bitter principle, which is said to be, not resinous, but of the nature of extractive.

"The aromatic principle of the Magnoliaceæ is found in many of their fruits; especially in the pericarps (seed cases) of the *Illiciums*, where it is very remarkable. These shrubs are known, under the name of Badian or star-Anis. The species which grows in China, (*I.* anisatum) bears stellated fruit, the odour of which is sweet, pungent, and aromatic, and its taste a little tart. The Chinese burn it in their temples; and the Europeans employ it, in rendering certain liquors aromatic, as for example, the Anisette de Bordeaux, from which we may conclude, that the aromatic principle of the stellated Anis has a resinous nature. A tincture of the fruit of the *Magnolia acuminata* is employed with some success in rheumatic diseases. The seed of almost every species of that genus are remarkable for their bitterness. This property is well known in the American species; and the seeds of the *M. precia* or *yulan*, known in China under the name of *Tsin-y*, are there employed as a bitter and febrifuge.

"Finally, the odour of the flowers of many species of this genus, has a decided action upon the nervous system. That of the *M. tripetata* or umbrella tree, causes nausea and headache; and Dr. Barton

* Drymis signifies in Greek, a pungent taste.

relates that the odour of the flowers of the *M. glauca* is so stimulating that he has seen it increase the paroxysm of a fever, and the pain in an attack of inflammatory gout. I should even cite on this occasion, the sweetness of the perfume afforded in India by the flowers of the Champacs, (*Michela*) in Japan by those of the Yulan (*Magnolia precia*, Corr. *M. Yulan* Desf.) and in Cayenne, by those of the *Magna odorata*, Aubl.—pp. 72—76.

"18.—VIOLACEÆ.

"The active properties of the Violaceæ reside in the roots, all of which appear to be endowed with emetic virtues, in a greater or less degree. Such are, among the species without reversed flowers, which constitute the genus *Pombalia* of Vandelli, or *Ionidium* of Ventenat, the *Viola parviflora*, *V. ipecacuanha*, and *V. itonbou* of Aubl. of which I have spoken in detail in my Memoir on the different kinds of Ipecacuanha;—such are also, among the true Violets, the *V. odorata*, *V. canina*, even the *V. tricolor*, the properties of which, although feeble, cannot be doubted. The last species, is distinguished, likewise, by the utility of its leaves and stems, in cutaneous eruptions, concerning which, however, new experiments are required.—p. 96.

"21.—HESPERIDEÆ.

"At the time of the first edition of this work, the family of Hesperideæ was so badly described in its limits, that it contained plants, heterogeneous in their properties as well as their forms; since that period M. Correa has asssigned the true boundaries of the family; and all the species which compose it are found to offer great uniformity, both in their structure and properties. The whole of these trees have wood of a remarkably compact texture; the bark and the leaves are furnished with a great number of vesicles filled with a volatile oil, aromatic, bitter, and stimulating; which gives to these parts decided tonic and exciting qualities; the rind of the fruit participates in these properties; the pulp is always more or less acid, as we see in the different kinds of Citron and in the *Limonia acidissima;* the same acid is found, though in a less degree, in the Oranges and Siam oranges. This acidity, and a little bitterness, renders these fruits refreshing, and useful in fevers and in scurvy. In the countries where the Hesperideæ are common, they use many different species indiscriminately.—pp. 99, 100.

"34.—CRUCIFEREÆ.

"All who have studied Botany, know how much the Crucifereæ present of uniformity, in their botanical, chemical, and medical characters; and hence it may, perhaps, be regarded as superfluous, to enter into details concerning them.

"The whole of the Crucifereæ, contain a very acrid volatile principle, for a long time supposed to be alkaline, and regarded by many chemists as formed in the plants themselves. It is correct to say, that, in their putrefaction, a considerable quantity, is disengaged; and that, by distillation, after fermentation, a certain portion is obtained; but neither their distilled water, nor their recently extracted juice, gives the least indication of alkalescence: we think, then, that ammonia does not exist, already formed, in the Cruciferæ, but that it is developed under certain circumstances, in consequence of the great quantity of azote, which these plants contain. It is probably to this azote, that we should refer the animal odour of the putrefying Cruciferæ; and, even, their great tendency to putrefaction; it is, perhaps, the necessity which they have for this principle, which causes them to grow, naturally, in such great numbers around the habitations of men and of animals.

"The acrid property of these vegetables, depends on a certain quantity of volatile oil; which may be extracted by different chemical processes, which possess the smell and taste of the Cruciferæ, and which is contained in small quantities, either dissolved or mixed, in their distilled water. This principle renders the Cruciferæ eminently stimulating: If it is concentrated, as we see it, for example, in mustard seed, and in the roots of the *Cochleariæ armoracia*, or, sometimes, in the herb itself, as in the *Lepidum latifolium*, then, the juice of these parts, applied upon the skin excites, first, a red ness, afterwards an active inflammation, and, in the end, an effusion of serum: this is the remedy known to every body under the name of sinapism.

"If this acrid and stimulating matter be administered, internally, it acts upon the nervous, and afterwards, upon the sanguiferous system, when it excites either diaphoresis, or, more frequently, diuresis. Given in large and repeated doses, it acts as an antiscorbutic; and it is for this purpose, that the Cruciferæ are most frequently and usefully administered. We know that the habitual employment of these vegetables, prevents scurvy, probably by sustaining the tone of the system; and that the same plants often cure scurvy already developed, either by re-establishing the strength or by acting as sudorifics or diuretics.

"When this acrid matter exists in a slight degree, these plants may serve as condiments, of which cresses are an example; when it exists, united with a considerable portion of mucilage or of saccharine matter, the mixture is highly nutritive, as in the cabbage, radish, turnip, &c. But this increase of nutritive matter does not annihilate their antiscorbutic properties, for a slight fermentation, which decomposes the saccharine matter, disengages the acrid principle, and restores these vegetables to the class antiscorbutics, of which the Sour krout is a striking example.

"When the plants of the order Cruciferæ have a taste too intense to be agreeable, we employ different precautions to obtain them more or less bleached, when they become more juicy and insipid: thus we are enabled to employ for food the roots or the lower parts of the stems, of many species, the leaves of which are neglected, such as the radish, turnip, &c. As in the Cruciferæ, which dispose their leaves into heads, as the cabbages, we choose, from preference, the central leaves, bleached and made tender by the absence of light: so we come, by different processes of cultivation, to make the flowers, in whole or in part, abortive, and thus to render the young shoots and the flower stems, a great deal more fleshy, and more mild and aqueous, as we see in the brocoli and cauliflower; sometimes we bleach, artificially, the stems, at the moment they sprout from the earth: it is by this culture that the English have made an agreeable pot-herb, of the sea-kale, so celebrated, formerly, among the Romans, as a coarse food reserved for the nourishment of the poor.

"The seeds of all the Cruciferæ contain a fixed oil, which, in many species, is so abundant, that it may be extracted with advantage, many examples of which we have, in the Colsa, Cameline, Rape, Julian, &c. When the fixed oil is mixed with a sufficient quantity of the pungent volatile oil, the seeds become, as I have already said, acrid, stimulating, diuretic, and sometimes anthelmintic.—pp. 111, 116.

"62.—UMBELLIFERÆ.

"Of all the families of plants, the medicinal characters of which we are rapidly tracing, there is not one which merits more scrupulous attention, than the umbelliferous; whether we refer to their dietetic and therapeutic importance, or to the anomalies which they present. In studying them, it will be necessary to attend with care, to the distinctions that exist among their organs, and even to those which their juices present.

"All the apparent anomalies of the order Umbelliferæ, seem to me to be explicable; by admitting that their extractive matter is narcotic, and their resinous principle, more or less stimulating and aromatic; or, in other words, that the sap, half elaborated, is narcotic, while, on the contrary, it becomes aromatic and stimulating when it is transformed into the true and proper juice. Let us follow the consequences, of this hypothesis, by applying it to each organ, in particular, and to the phenomena, in general, which the plants of this order present.

"Vegetable physiology teaches us, that the moisture of the soil enters the plant through the extremeties of its radicles; that it ascends through the body of the roots, and then mounts through the trunk to the branches; where, by an unknown chemistry, it is transformed into the proper juice, and returns to the root along the bark. The root then contains a great quantity of sap, not yet elaborated; and a certain quantity of proper juice, which has redescended through the bark; consequently, according to our hypothesis, the roots of the Umbelliferæ, should contain an aqueous mucilage, more or less insipid; and more or less aromatic, from the presence of the proper juice; they ought not to be dangerous, since they contain little or no extractive, and may, therefore, be edible and nourishing, for man; as we find to be the case, with the roots of the carrot, parsnip, angelica, eryngo, &c., &c.; among which we only observe differences, more or less considerable, in the quantity of the aromatic, saccharine principles. The roots even of the poisonous Umbelliferæ, are sometimes salubrious; of which we have a curious example, in the *Œnanthe pimpinelloides*, the tuberous roots of which serve as food at Angers, under the name of *Jouanettes*, and at Saumur, under that of *Mechons*. Many of the Umbelliferæ, present, in their roots, a notable quantity of saccharine matter; thus according to M. Drapier, there is fourteen per cent. in the dried carrot, twelve and a half per cent. in the parsnip, and eight per cent. in the skirret. In the herb, on the contrary, we find a large quantity of extractive, mixed with, or dissolved in the sap; and which we obtain by infusion or decoction, in water. From the bark we may procure, a variable quantity of proper juice, more or less aromatic, or more or less resinous; consequently, in the natural state of things, the extract of the umbelliferous plants, ought to be narcotic, as we find it in the *Conium maculatum*, the *Cicuta virosa*, the *Æthusa cynapium*, &c., while, on the contrary, the proper juice, extracted from the bark, either by an incision, or by pharmaceutic manipulations, should be tonic, stimulating, or aromatic, as we see it in the *Galbanum*, *Opopanax*, Lovage, *Assafœtida*, &c. Further, if we employ, at different times, the bark and trunk, that is to say the sap and the proper juice united, the qualities of the mixture must vary, according to the proportions of the two principles.

"Finally, if we use the seeds, as we find in them, not only the sap, (at least when they have come to maturity) but a liberal quantity of volatile oil, lodged in their exterior integument, we ought to regard them as free from poisonous qualities, and endowed with aromatic, stimulating, and tonic powers; and in fact, these properties are common to the seeds of the whole of the Umbelliferæ in all countries.

"Let us approach the subject still closer, and we shall find, that the hypothesis, which I have stated above, explains even the variations which soil, age, and culture, occasion in the properties of the Umbelliferæ. Thus we know, in general, that aquatic plants contain, proportionably, a greater quantity of sap, than of proper juice; more mucilage and extractive, than oil and resin; therefore we should not be surprised, to see the Umbelliferæ more narcotic, when they grow in wet, and more hot and aromatic, when they vegetate in dry places; we may even conceive how those which are bleached, contain a sap scarcely elaborated, and might thus be assimilated to the roots of these plants; while, on the contrary, those which are exposed to a strong light, contain a larger proportion of proper juice.

"According to these considerations, I think that the Umbelliferæ conform to the laws of analogy, as I understand them; that is to say, that each juice and each organ, preserves the same nature and the same properties, in each family. I will add, that the organ which furnishes the true botanical character of the family, viz. the seed, is that in which Chemistry and Medicine find the fewest anomalies.—pp. 159—163.

"94.—LABIATÆ.

"The Labiatæ constitute, perhaps, the most natural family to be found in the vegetable kingdom. The resemblance in their forms is such, that no botanist has yet attempted to disunite them; and it is, indeed, difficult to separate them into secondary groups or genera. The properties of these plants offer a resemblance quite as striking.

"All the Labiatæ are remarkable for their cordial, stomachic, and tonic virtues; we may distinguish, in the whole of them, according to the observations of M. de Jussieu, two principles—the one bitter, the other aromatic,—mixed in varying proportions. Their bitterness, which is communicated to hot water, appears to reside in a gum-resinous principle, which is found, more or less abundantly in every species; those in which it *abounds*, as the Scordium (*Tenerium scordium*), the Germander (*T. Chamadris*), &c., are particularly employed as stomachics; and even sometimes, as febrifuges; those, on the contrary, which are abundant in volatile oil, and which are, consequently, more aromatic, are used as heating, and exciting stimulants; the different mixtures of these two principles, and the particular state of each, have led to the designation of a great number of these plants to particular uses, in medicine and dietetics; some serving as condiments for our food—such as Marjoram, Summer Savory, Sweet Basil, &c., the others, furnishing by infusion, a slight tonic drink, which we take as tea; such as Sage, Balm, &c. Several Labiatæ reduced to powder, are employed as sternutatories and cephalics; for example, Marum, Marjory, and Lavender; others, as Thyme, Serpillum or wild Thyme, &c., are used as perfumes; while others furnish the distilled waters, of which so great a quantity is used, such as Balm, Lavender, Mint, and Rosemary named, improperly, the distilled water of the Queen of Hungary.

"All of them can furnish a quantity, more or less considerable, of volatile oil; a product which is most remarkable, in the Thymes, Origanums, and Lavenders. Finally, the chemists have increased our knowledge of the uniformity of the composition of the Labiatæ, by showing to what a great extent they abound in Camphor. It was long ago remarked, that many of them exhaled the odour of that drug, and some crystals of it had been found by Gaubius, in the oil of Thyme, by Kunkel in that of Rosemary, by Kruge in that of Marjoram, by Cartheuser in that of Wild Thyme, &c. and M. Proust has lately proved, that it exists, even in quantities so great that it might be extracted with advantage, in the oils of Sage and Lavender; and, probably, in all the distilled oils of this order of plants.—pp. 231—234.

We might quote many other, and perhaps more interesting articles, but wish not to tire the patience of the reader. What we have laid before him, must produce the conviction, that plants which resemble each other in their botanical habit, also resemble one another, in their medical, economical, or dietetic properties; the proposition which our learned and ingenious author proposed to establish.

The third and last part of his Essay is brief. It embraces a tabular view of the orders, with a statement of their conformity or nonconformity to the law of analogy; we have not room for its insertion, and must content ourselves with a summary of general results:

"Of the 150 natural orders, known to botanists, there are,' says our author,

"Forty, which are either inert, or the properties at the present time, unknown.

"Twenty two, in which we may presume the law of analogy to exist, although we are, as yet, acquainted with the properties of but a small number of the species.

"Twenty, in which we must admit the law of analogy to be restricted to certain genera, while others are widely separated from them, by important characters.

"Thirty five, in which the law of analogy is evident, but still they present some exceptions.

"Thirty one, in which the law is entirely preserved.

"Three, in which it is violated; but in which we still find undoubted traces of its existence.

"Thus, the law of analogy between the forms and properties, is more or less true, in one hundred and nine orders, and scarcely false in three.

"I think, then, we may draw from this dissertation the following conclusions.

"1. The same parts, or the corresponding juices, in plants of the same kind, enjoy similar medical properties.

"2. The same parts, or the corresponding juices, in plants of the same natural order, possess analogous properties.

"3. The apparent exceptions to these two laws, arise from one or more of the following causes:

*a.* From the difference in the degree of distinction, real, although not always recognized in botanical works, between the species of a genus, and the genera of a natural order.

*b.* From a false comparison between the organs of similar plants.

*c.* From the accidental, or temporary state, in which certain vegetables are, at the period when we are accustomed to employ them.

*d.* From the unequal mixture of different chemical principles, common to all analogous plants.

*e.* From differences in the mode of extraction or of preparation, a circumstance which modifies the nature of medicines.

*f.* From this, that we are apt to attach too much importance to properties which are purely accidental.

*g.* From this, that we do not compare with accuracy, the modus operandi of different medicines.

*h.* From this, that we do not examine, comparatively, the mode of application of medicines to the human body.

"4. Analogy (founded upon the probability of 109 against 3) leads us to think, that the orders constituting exceptions, which in the present state of the science, cannot be explained away, will come under the laws which we have laid down, when Medicine, Chemistry, and Botany, shall have attained to greater perfection. This is at least extremely probable, seeing, that since the first edition of this work, ten years ago, the number of exceptions has been greatly diminished. At that time, the law was only founded on the comparison of 85 to 7, while it now rests upon the relation of 109 to 3, or triple the former proofs."

With these extracts, we close our account of De Candolle's book. As far as we know, it has not before been presented to the notice of the profession, in America; and in our own language, we have not met with a single distinct and formal treatise, on the subjects which it embraces. The analysis, therefore, with all its imperfections, will not, we trust, be unacceptable to the majority of readers.

To make known the plan and merits of this work was one of our objects; the other was to recall the attention of the physicians of the United States to the cultivation of Botany. Twenty years ago, this delightful science was more attended to by them, than at present. This was chiefly owing to the late professor Barton; who might justly be styled the father of American Medical Botany. His "Collections" are repeatedly quoted by our author, and his "Elements of Botany" were republished in England. We should like to see them in the hands of every student of medicine in the United States. After some acquaintance with the elementary works of Lee, Smith, and Willdenow, we do not hesitate to say, that, for the American student in Botany, they are preferable to either. Published nearly a quarter of a century ago, and little known to the majority of our readers, we might, without editorial impropriety, have made them the subject of a Review, designed to recommend the cultivation of Botany; but, in addressing ourselves to medical men, we preferred, as a text, a work professedly designed to illustrate one of the most important connexions between Botany and Medicine.

In regard to books on American Botany, a few remarks may not be out of place. Much, indeed most, of what has been written on the Botany of this country, has been by foreigners; and their works, generally, are not within the reach of the students of the United States. Not to go further back than to the *Flora Boreali Americana*, of the elder Michaux, we may say, that scarcely any book of science is more seldom met with in this country. The *Sylva Americana*, of his son, recently translated, is oftener seen in our booksellers' shops, and the plates which embellish it are of the first order of excellence; but, as its name imports, it is limited to our forest trees; and, in reference to them, is rather economical than botanical. It is not, in short, the kind of practical book which the student requires. The next in order of time is the *Flora Americæ Septentrionalis*, of Pursh, in 2 vols. 8vo, published in 1814. It embraces the general Botany of the United States, and professes to give a description of all the known American plants, up to the period at which it was written. Of course, it contains many species, and a number of genera, not before embodied in any systematic work. The author, a German, resided long, and travelled to some extent, in the United States, especially east of the mountains; and his work was, therefore, a substantial addition to our *Bibliotheca Botanica*, but still it seems to have attracted no great degree of attention. It is mortifying to be compelled to suspect, that the circumstance of his specific descriptions being in the Latin tongue, has limited the circulation of his book among the medical profession of this country! To Pursh's book succeeded the *Genera of North American Plants* of our friend Mr. Nuttall, at present, the keeper of the Botanic Garden, attached to Harvard University. It contains a description of the genera, and a catalogue (not descriptive) of the species, known up to 1817. Mr. Nuttall is an Englishman by birth, but has resided long in the United States; and no botanist has ever explored them (particularly the western, almost to the Chippewan Mountains) with a more untiring step. His book is cheap, and should be in the hands of every student of American Botany. With it, and the practical work of Mr. Pursh, and the Elements of Professor Barton, no young man of industry and observation, could be long, in acquiring a competent knowledge of the plants which might surround him.

The works of the two Michauxes, of Pursh, and of Nuttall, are *general*, and we have, therefore, recommended them; but there are others of great merit, which relate to particular districts, and should be in the possession of the students who reside in them, respectively. Of this kind, to quote from memory at the moment, are the *Flora Lancastriensis* of the late Dr. Muhlenburg; the *Flora Philadelphiensis* of Dr. W. P. Barton; the *Florulæ Cestrica* of Dr. Darlington, a pupil of Professor Barton, the *Florula Bostoniensis* of Professor Bigelow, in the opinion of the late Abbe Correa, destined to be the Linnæus of America, but who seems to have abjured the practice of Botany; Dr. Torrey's Botany of the Northern States, and Mr. Elliot's Flora of South Carolina—all published in the United States, and, therefore, within the reach of its students and physicians.

Of foreign systematic works, the best for the American student, is Turton's edition of the Systema Naturæ of Linnæus; but it can by no means be substituted for those which have been named.

We may be asked, however, whether it will not be sufficient for physicians to obtain and study the writings of Woodville, Barton, and Bigelow, on *Medical* Botany? We answer,

No. These are valuable works, and the two latter, especially, should be in the library of every American physician; but he cannot study them without an acquaintance with the elements of Botany, nor with such an acquaintance can he become a medical botanist, without first acquiring some knowledge of general Botany.

To this end he should collect and arrange an *Herbarium* or *Hortus Siccus* of the plants within his reach, an undertaking of easy and most progressive accomplishment. The observance of but a few rules is necessary. We may state the most important.

1. Collect a specimen in flower, and whenever practicable, another in fruit.

2. When the fertile and infertile flowers are seated on different plants, or different parts of the same plant, collect specimens of both.

3. Place the specimen, before it has *wilted**, between leaves of paper (the finer its texture, the more beautiful will be the appearance of the specimen) and subject it to pressure for 24 hours. Then place it in a fold of fresh paper of a common quality, and remove it from day to day, until it has become dry. During the period of exsiccation a gentle pressure only should be employed.

4. When thus prepared, the specimens representing a species, should be fastened with slips of paper and paste to the third page of a sheet of white paper; on which should be inscribed the class, order, genus, and species of the plant, in the method of Linnæus, and, if possible, its natural order. To

* We beg pardon of our brother criticks, for employing a word not to be found in the English nor even in the American classicks. *Wilted*, however, is neither a new word nor an Americanism; and although not generally used by our writers, deserves to be. A wilted plant is in a state of suspended animation or a kind, of fainting fit: a withered plant is actually dead and dry. The former can be revived; the latter cannot. The two states differ widely in their external symptoms, as well as their internal constitution, and cannot, therefore, be expressed or described by the same word. The young plant transferred from the spot where it germinated, is *wilted* by the meridian sun; but the following night it may recover its erect position;—if it should not in three days, it will be *withered*.

which should be added its locality, times of frondescence and flowering, and its popular name.

5. The species should now be arranged into genera, within the duplicatures of larger papers, and these bundles disposed into orders and classes, on the shelves of an *Herbarium*, in a dry situation. In periods of great humidity, it will be necessary to expose them to the open air, to prevent their moulding. The *acarus* and other minute insects which prey upon them, may be repelled or destroyed by camphor, or a solution of the deuto chloride of mercury, in distilled or rain water.

It is unnecessary to add other instructions; and, admonished, by a feeling of weariness, of what our readers are likely to suffer, if longer detained, *in sylvis tenebrosis*, we shall now permit them to escape.

